# Evaluation of Heavy Metal Contamination in Paddy Plants at the Northern Region of Malaysia Using ICPMS and Its Risk Assessment

**DOI:** 10.3390/plants10010003

**Published:** 2020-12-22

**Authors:** Nur Syahirah Zulkafflee, Nurul Adillah Mohd Redzuan, Jinap Selamat, Mohd Razi Ismail, Sarva Mangala Praveena, Ahmad Faizal Abdull Razis

**Affiliations:** 1 Department of Food Science, Faculty of Food Science and Technology, Universiti Putra Malaysia, 43400 UPM Serdang, Selangor, Malaysia; nursyahirahzulkafflee@gmail.com (N.S.Z.); dillaredzuan94@gmail.com (N.A.M.R.); sjinap@gmail.com (J.S.); 2 Laboratory of Food Safety and Food Integrity, Institute of Tropical Agriculture and Food Security, Universiti Putra Malaysia, 43400 UPM Serdang, Selangor, Malaysia; smpraveena@upm.edu.my; 3 Laboratory of Climate-Smart Food Crop Production, Institute of Tropical Agriculture and Food Security, Universiti Putra Malaysia, 43400 UPM Serdang, Selangor, Malaysia; razi@upm.edu.my; 4 Department of Environmental and Occupational Health, Faculty of Medicine and Health Sciences, Universiti Putra Malaysia, 43400 UPM Serdang, Selangor, Malaysia; 5 Natural Medicines and Products Research Laboratory, Institute of Bioscience, Universiti Putra Malaysia, 43400 UPM Serdang, Selangor, Malaysia

**Keywords:** average daily dose, enrichment factor, hazard index, lifetime cancer risk, paddy soil, translocation factor

## Abstract

Heavy metals from natural and anthropogenic sources accumulate in soil and plants and as a consequence represent important environmental contamination problems. Nevertheless, food safety issues and adverse health risks make this one of the most serious environmental issues. The aim of the present study was to assess heavy metal contamination in the paddy plants from the northern area of Malaysia using Inductively Coupled Plasma Mass Spectrometry (ICPMS) and its risk assessment. In total, the heavy metals (As, Cd, Cu, Cr, and Pb) of the samples of paddy plants harvested from Kedah areas were extracted using an acid digestion method, while the heavy metals for soil samples using ammonium acetate. The heavy metal concentrations were then analysed using Inductively Coupled Plasma Mass Spectrometry (ICP-MS). The enrichment (EF) and translocation factors (TF) of heavy metals were calculated, and health risk assessment (HRA) was performed. The EF values for heavy metals from the soil to roots, roots to stems, stems to leaves, and stems to grains followed the order Cu > As > Cr > Cd > Pb, whereas Cr and Pb were characterized by greater TF values from stem to grain than the other elements. The average daily dose (ADD) for both children and adults is below the safe value intake for each of the studied elements. The combined hazard index (HI) of five elements was beyond the acceptable value (HI >1). The carcinogenic risk, as exemplified by lifetime cancer risk (LCR), indicated that single exposure to As or Cr, in both adults and children, was greater than 10^−4^. The total cancer risk (CRt) resulting from multiple exposure to carcinogenic elements exceeded the acceptable value (CRt >1 ×10^−4^) in both adults and children. Overall, exposure to heavy metals through rice consumption poses potential non-carcinogenic and carcinogenic health risks to the local residents in the northern area; thus, regular monitoring of pollution in the area is crucial.

## 1. Introduction

Rice belongs to the genus *Oryza* which is known as one of the complex carbohydrates that plays significant role as a source of energy but also contains moderate amounts of protein. Rice is also rich in various types of micronutrients such as vitamin B, thiamine, riboflavin, and niacin [1,2]. According to the International Rice Research Institute [3], there are many varieties of rice found around the world. In Malaysia, there are eight varieties of rice marketed according to Padiberas Nasional Berhad (BERNAS) Malaysia, these being parboiled rice, glutinous rice, basmati rice, local white rice, fragrant rice, broken rice, brown rice, and imported white rice. Since rice supplies almost half of the daily calories of the world’s population, it has been highlighted as one of the most important foods in the world [4,5,6,7]. Meanwhile, in Malaysia, rice is recognized as the third most dominant agricultural food product [8]. 

Rice is known as the staple food of more than half of the world’s population, with more than 3.5 billion people relying on rice consumption for their daily calories. Rice has contributed almost 19% of global human per capita energy and 13% of per capita protein. Asian countries particularly account for 90% of global rice consumption, with total rice demand continuing to escalate. On the other hand, outside Asia, where rice is not a staple food, per capita consumption continues to rise. Rice is the fastest growing staple food in Africa, as well as Latin America. Globally, rice consumption remains sturdy as determined by both population and economic growth, especially in many African and Asian countries [9]. Zavala and Duxbury [10] reported that the daily intake of rice in Asian countries reached 0.5 kg (dry weight) per capita. As the population continues to grow and consequently rise demand in rice consumption, producers employ escalating amounts of pesticides in order to protect their crops [11,12]. Thus, heavy metal pollution in paddy fields has been recently of great concern as an environmental pollutant because of bioaccumulation in the environment and the non-biodegradable properties of the metals. 

Heavy metals have been frequently discussed as potential pollutants in rice [13] and are classified major toxic chemicals due to their high potential risk to the ecosystem and human health. By nature, heavy metals can be divided into two forms namely organic and inorganic [14]. The International Agency for Research on Cancer (IARC) has classified inorganic heavy metals as group 1 carcinogens, since long-term exposure is associated with an increased risk of various types of cancer [15]. In contrast to the inorganic pollutants, heavy metals in the organic form may gradually degrade to less harmful products through chemical or biological processes [16]. Environmental contamination of the biosphere with heavy metals caused by intensive agricultural and other anthropogenic activities poses critical problems for safe use of agricultural land [17]. Agricultural soils are potentially contaminated with essential and nonessential heavy metals through current agricultural practices including the indiscriminate use of agrochemicals such as pesticides and fertilizers along with mechanical cultivation [18,19]. Pesticides and heavy metals are the most hazardous contaminants of agricultural soils. In fact, the agricultural sustainability has long been associated with the use of a broad spectrum of pesticides that control the disease-causing pests and crop destroying insects [18,19]. 

The accumulation of heavy metals in agricultural soils is a growing concern to the public as well as government agencies, due to the food safety issues and potential health risks as a result of its detrimental effects on soil ecosystems [20]. International agencies, such as the Food and Agriculture Organization (FAO) and the World Health Organization (WHO), are currently advocating compliance to permission criteria of pollutants in agricultural products [21]. Heavy metals are among the major contaminants in food supply [22]. Heavy metals such as arsenic, cadmium, and mercury are of primary concern in soil and food contamination, particularly in rice cropping systems, because of their high toxicity that pose major human health risks through the dietary intake of food crops contaminated by root transfer from soil to plant tissues or direct atmospheric deposition onto plant surfaces [23]. These toxic elements accumulate in the soils, induce a potential contamination in the food chain, and endanger ecosystem safety and human health [20]. 

Every metal and plant interact in a specific way, which depends on several factors such as soil type, plant, growth conditions, and the presence of other ions. Metal uptake by grains was directly related to the applied heavy metal with greater concentrations of metals found in cases where metals were added separately rather than in combinations. Different tillage systems and agricultural crops rotation affect the uptake and distribution of heavy metals. Liu et al. [19] reported that rice plant and vegetables accumulated heavy metals from the agricultural soil under actual natural condition. Plant roots take up metal contaminants and/or excess nutrients from growth substrates through rhizofiltration (=root) process, the adsorption, or precipitation onto plant roots or absorption into the roots of contaminants that are in solution surrounding the root zone. The plants act both as “accumulators” and “excluders”. Accumulators survive despite concentrating contaminants in their aerial tissues. They biodegrade or biotransform the contaminants into inert forms in their tissues. The excluders restrict contaminant uptake into their biomass [24].

Plants have evolved highly specific and very efficient mechanisms to obtain essential micronutrients from the environment, even when present at low concentration levels. Plant roots—aided by plant-produced chelating agents and plant induced pH changes and redox reactions—are able to solubilize and take up micronutrients from very low levels in the soil, even from nearly insoluble precipitates. Plants have also evolved highly specific mechanisms to translocate and store micronutrients. These same mechanisms are also involved in the uptake, translocation, and storage of toxic elements, whose chemical properties simulate those of essential elements [24]. It was also reported that heavy metals including cadmium were accumulated in different rice cultivars [24]. The various body parts of crop plants differ in their ability to absorb and accumulate heavy metals as well as in the metal uptake and translocation between plant species and even between cultivars of the same plant species [24,25,26,27,28,29,30,31]. Arao and Ac [26] revealed that cadmium levels in the rice grain were also influenced by genotypic variations which leading to the uptake and distribution of cadmium in different rice cultivars [27]. Another factor that influences the absorption and accumulation of heavy metals is the concentration of organic matter in the layer soil. The direct transfer of heavy metals to the human body from plant parts is of interest in the case of rice grain, which is extensively consumed, thus posing a threat to human health [32]. 

The accumulation of heavy metals in paddy plants may be the consequence of overuse of various types of fertilizers and pesticides for their cultivation in Malaysia, especially in Peninsular Malaysia, and is an issue of great concern. Since rice is a staple food for most of the world including Malaysia, it is important to determine the content and mechanism of heavy metal uptake in paddy soils and plants. In Malaysia, the study regarding the heavy metal content in paddy plants and soils has not been well documented, and there is lack of information regarding the enrichment and translocation factors in paddy plants and soils. Therefore, the main objective of the present study was to analyse the heavy metal content in paddy soils and paddy plants from the northern area of Malaysia, and to increase our understanding of the underpinning mechanism of heavy metal uptake through the calculation of the enrichment (EF) and translocation factors (TF) in accessing the accumulation of heavy metals in rice grains.

## 2. Materials and Methods 

### 2.1. Sampling Area

**Figure 1:**
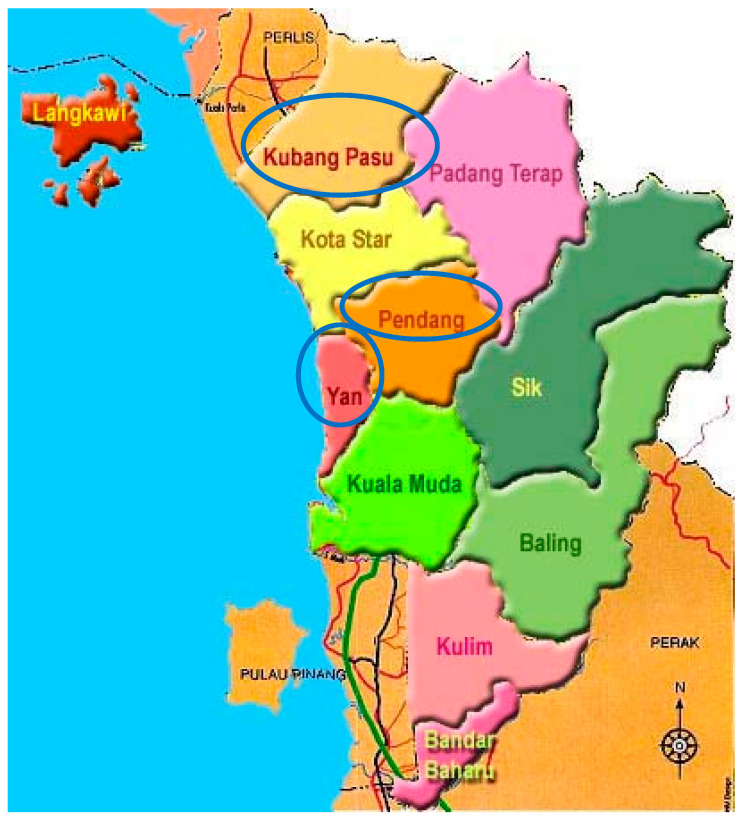
Map of sampling areas of paddy in Kedah, Malaysia Northern region.

The paddy field sampling sites from Kedah, namely Yan, Kubang Pasu, and Pendang (Figure 1), were representative of the north region in Peninsular Malaysia within the area from latitude 5° 47’ 59.99” N to 6° 16’ 5.23” N and from longitude 100° 21’ 59.99” E to 100° 27’ 59.99” E. Kedah was chosen because this state is among the major rice producing areas of Malaysia. Three paddy planting areas, which plant MRQ 74 and MR 219 varieties, were selected randomly at each sampling site. At each area, three plots of 1000 m^2^ were randomly selected for sampling; the sampling frequency was thrice at each sampling plot. The sampling was conducted at growth stage #8 (code 89), in which the rice plant was fully ripened in the paddy field. For soil, samples were taken from 0 to 30 cm depth with a soil hand auger. Several paddy plants were uprooted from the soil at each plot and stored in clean plastic bags. The sites of the study were labelled for each sample we collected as YP1, YP2, and YP3 for Yan plots 1, 2, and 3, respectively; KPP1, KPP2, KPP3 for Kubang Pasu plots 1, 2, and 3, respectively; and PDP1, PDP2, and PDP3 for Pendang plots 1, 2, and 3, respectively.

### 2.2. Soil Preparation and Extraction

Sample preparation was carried out as described by Khairiah et al. [8] with slight modifications. The soil samples were dried at room temperature before being ground and passed through a 0.25 mm mesh sieve (No. 60 mesh sieve), prior to analysis. Each sample from each plot area was prepared in triplicate. In the case of soil samples, the heavy metals were extracted using ammonium acetate, at pH 7 [33]. Ten grams of soil were weighed into a conical flask, 50 mL of 1 M NH_4_CH_3_OO (pH 7) was added, and the mixture was shaken for 1 ½ hours, followed by centrifugation at 3000 rpm for 30 min. Finally, the samples were filtered through a 0.45 µm Millipore filter paper using a syringe. 

### 2.3. Paddy Plants Preparation and Extraction

The paddy plants were washed under running tap water followed by two rinses with distilled water and one rinse with deionized distilled water. Subsequently, the samples were dried with clean tissue, cut, and separated into the whole parts of leaves, stems, roots, and grains. After separation, the various parts of the paddy plant were dried in an oven (Memmert Oven, Schwäbisch Hall, Germany) at 65 °C for 48 h. Dried leaf, stem, and root were ground using a blender, whereas the dried grains were ground into powder using a pestle and mortar and sieved using a 0.25 mm mesh sieve (No. 60 mesh sieve). Each sample from each plot area was prepared in triplicate. Heavy metals were extracted from the paddy plant parts using an acid digestion method [34]. An aliquot (1 g) was placed in a 100 mL conical flask and 10 mL of 69% nitric acid (HNO_3_) was added. Digestion was performed on a hot plate at 60 to 80 °C until the brown gas evaporated, and 60% perchloric acid (5 mL) was added until the mixture became clear. The cooled mixture was filtered through a 0.45 μm Millipore filter using a syringe before being transferred into a 50 mL volumetric flask and made up to the mark with distilled water.

### 2.4. Sample Analysis

Heavy metal (Pb, Cd, Cu, Cr and As) analysis was performed using Inductively Coupled Plasma-Mass Spectrometry (ICP-MS) (Perkin Elmer Elan DRC-e, Santa Clara, CA, USA). As to uphold the quality, 10% nitric acid (HNO_3_) was employed to soak all the lab equipment. A standard solution was run together with the blank sample in every 10th sample, and the rice samples were analysed in triplicate together with Certified Reference Material (CRM) IRMM 804 in the range of 90.5% to 102.4%.

### 2.5. Enrichment Factor (EF) and Translocation Factor (TF)

The food chain pathway (soil–plant–human) is known as one of the vital pathways for human exposure towards soils contamination. The transfer of soil-to-plant is among the significant processes of human exposure to toxic elements via the food chain. The equations used for the calculation of the enrichment [35] and translocation [36] factors are shown below. The value of EF indicates the mechanism of heavy metals absorption from soil to paddy grain, whereas the value of TF examined the transport or transfer of the heavy metals from soil to other parts of the paddy plant (root, stem, grain) (Equations (1) and (2)).

Enrichment Factor (EF) = Cp/Cs(1)

where Cp represents the heavy metal concentration in the grain and Cs in the soil.

Translocation Factor (TF) = Cq/Cr(2)

where Cq represents the heavy metals concentration in the edible part of plant and Cr in the plants’ soil or root or stem.

### 2.6. Human Risk Assessment (HRA)

The HRA values including hazard quotient (HQ), hazard index (HI), and lifetime cancer risk (LCR), were calculated based on the equation provided by USEPA 2012 [18]. The typical rice Ingestion Rates (IR) used for adults and children were 600 g/day and 198.4 g/day, respectively (Equations (3) and (4)).

HQ = ADD/RfD(3)

where ADD represents the average daily dose, and RfD is the reference dose, which is an index of the estimated maximum permissible dose for humans through daily exposure.

(4) $$\mathrm{HI}\;=\sum^{\;}\mathrm{HQ}$$

where HI < 1 indicates that chronic risks are unlikely; HI > 1 constitutes that noncancerous risks are likely to occur.

### 2.7. Statistical Analysis

Statistical analysis of the data was performed using Minitab version 17.0 by one-way analysis of variance (ANOVA) and Tukey’s test.

## 3. Results and Discussion

### 3.1. Heavy Metals Concentration in Paddy Soil

Heavy metal concentrations in paddy soils for the three plots of Yan, Kubang Pasu, and Pendang are shown in Table 1. Chromium (Cr) showed the highest concentration followed by lead (Pb), arsenic (As), copper (Cu), and cadmium (Cd) in Yan (YP1, YP2 and YP3) and Kubang Pasu (KPP1, KPP2, and KPP3) areas. Meanwhile, Pb showed the highest concentration in the Pendang area (PDP1, PDP2, and PDP3) followed by Cr, As, Cu, and Cd, respectively. Hence, lead and chromium were the most abundant heavy metals of those studied areas in all nine samples of paddy soils, while cadmium was the lowest probably due to the proximity to Straits of Malacca in the case of Yan and Kubang Pasu. However, all elements (Cu, Cd, Cr, Pb, and As) in paddy soils for all studied areas were below the maximum allowable concentration based on standards recommended by Chinese Environmental Quality Standard for Soils, grade II [37], and European standards agriculture soils [38]. The lowest concentration of Cd in paddy soils was consistent with the previous studies reported in Malaysia [8,20,39,40,41,42].

### 3.2. Heavy Metal Concentration in Paddy Plant Parts

The concentrations of heavy metal in paddy plant parts (roots, stems, leaves, grains) of the Yan area are presented in Table 2, Kubang Pasu area in Table 3, and Pendang area in Table 4, respectively. In Yan area, the concentration of Cu was the highest in roots, stems, leaves, and grains. Therefore, heavy metal concentrations accumulated in grains for the Yan area can be presented in descending order as follows: Cu > Cr > As > Pb > Cd. Different results were found in the Kubang Pasu area, whereas Pb concentration was the highest in roots, and Cr concentration was highly accumulated in grains. Moreover, the highest concentration of arsenic was found in roots and grains, while copper in stems and leaves for samples of the Pendang area. However, all elements (Cu, Cd, Cr, Pb, and As) in paddy grains for all studied areas were below the maximum allowable concentration based on standards recommended by Malaysian Food Regulation 1985 [43] and CODEX standard [44].

Food Regulation 1985 ^A^

1000

400

2000

**-**

**-**

CODEX standard ^B^

200

400

200

20,000

-

The heavy metal accumulation in rice might be affected by physical–chemical properties of soil growing as a function of soil concentration. The equilibrium of pH value could influence the sorption and desorption of heavy metals towards soil components and thus is related to the ability of metal transfer. The more acidic the soils, the greater the heavy metal transfer from soil to rice. Results in the present study revealed that all the metals studied were present in all parts of *Oryza sativa* at different levels. In general, the heavy metals were accumulated mostly in paddy parts of root compared to stem, leaf, and grain.

### 3.3. Enrichment Factor (EF)

**Figure 2:**
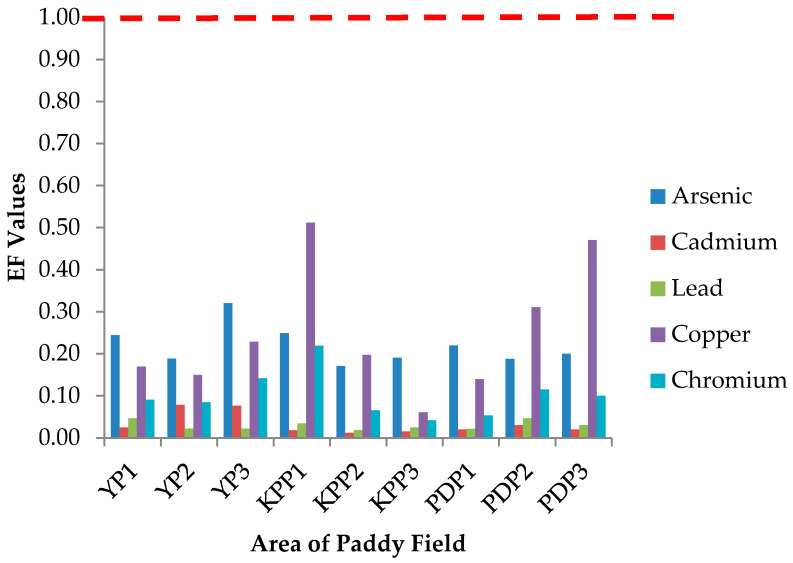
Enrichment factor (EF) from the soil to grain of paddy in areas of Yan, Kubang Pasu, and Pendang. YP1: Yan Plot 1, YP2: Yan Plot 2, YP3: Yan Plot 3, KPP1: Kubang Pasu Plot 1, KPP2: Kubang Pasu Plot 2, KPP3: Kubang Pasu Plot 3, PDP1: Pendang Plot 1, PDP2: Pendang Plot 2, and PDP3: Pendang Plot 3.

The enrichment factor (EF) and translocation factor (TF) can be used to analyse the absorption mechanism of metals in paddy plants. The soil-transfer-rice factor or EF is an index to evaluate the potential of a metal transfer from soil to plant. Figure 2 shows the enrichment factor from the soil to the edible parts of paddy which is grain emanating from the Yan, Kubang Pasu, and Pendang areas. The EF values for all studied metals were lower than 1 in the ranking order of Cu > As > Cr > Cd > Pb. If EF < 1 or EF = 1, the value denotes that the plant only absorbs but does not accumulate or store heavy metals, whereas if EF > 1, the value represents that plants are able to accumulate the metals. Among the investigated metals, EF values in KPP1 were found to be the highest for Cu (0.51), which were obtained from the heavy metal concentration of grain (175.98 µg/kg) divided by the heavy metal concentration of soil (345.06 µg/kg), whereas in KPP2, were found to be the lowest for Cd (0.01). The low EF value of Cd in KPP2 was inconsistent with previous findings where Cd was characterized with higher mobility from soil to rice grain, so that soil contaminated with Cd would pose a major health risk [45,46]. The discrepancy may be due to the relatively low Cd concentrations in soil and grain in the present study as well as the climatic conditions which could play an important role in the movement of minerals and metals. The high Pb content in soil does not lead to accumulation in rice grain.

### 3.4. Translocation Factor (TF)

**Figure 3:**
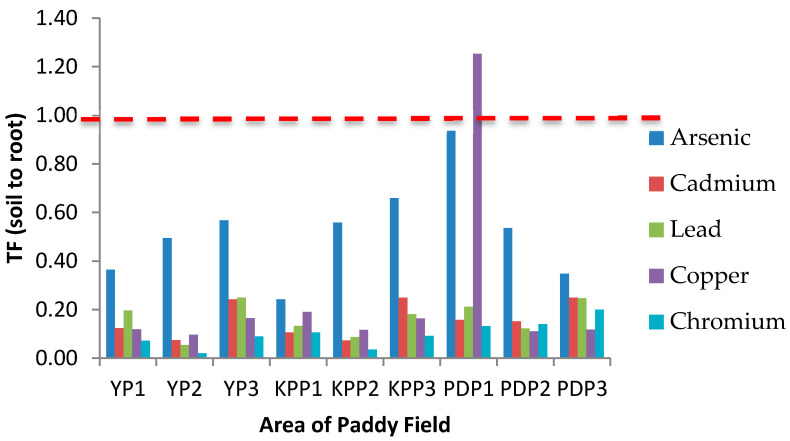
Translocation factor (TF) of studied metals from soil to root (TF_Soil_) in areas of Yan, Kubang Pasu, and Pendang. YP1: Yan Plot 1, YP2: Yan Plot 2, YP3: Yan Plot 3, KPP1: Kubang Pasu Plot 1, KPP2: Kubang Pasu Plot 2, KPP3: Kubang Pasu Plot 3, PDP1: Pendang Plot 1, PDP2: Pendang Plot 2, and PDP3: Pendang Plot 3.

**Figure 4:**
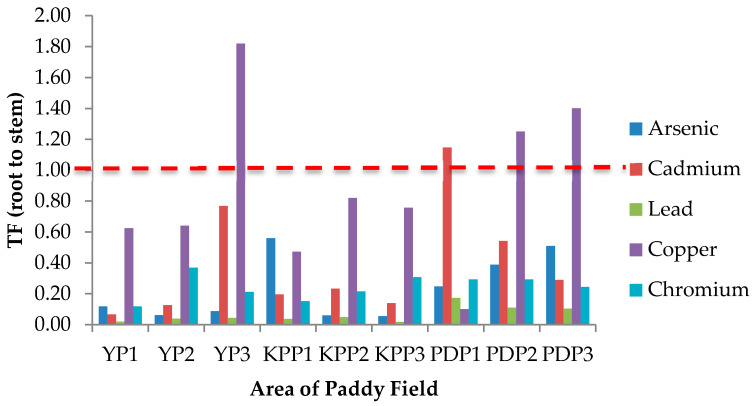
Translocation factor (TF) of studied metals from root to stem (TF_root_) in areas of Yan, Kubang Pasu, and Pendang. YP1: Yan Plot 1, YP2: Yan Plot 2, YP3: Yan Plot 3, KPP1: Kubang Pasu Plot 1, KPP2: Kubang Pasu Plot 2, KPP3: Kubang Pasu Plot 3, PDP1: Pendang Plot 1, PDP2: Pendang Plot 2, and PDP3: Pendang Plot 3.

**Figure 5:**
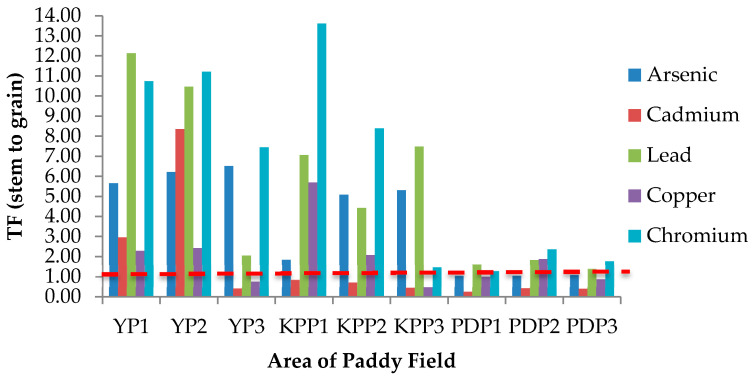
Translocation factor (TF) of studied metals from stem to grain (TF_stem_) in areas of Yan, Kubang Pasu, and Pendang. YP1: Yan Plot 1, YP2: Yan Plot 2, YP3: Yan Plot 3, KPP1: Kubang Pasu Plot 1, KPP2: Kubang Pasu Plot 2, KPP3: Kubang Pasu Plot 3, PDP1: Pendang Plot 1, PDP2: Pendang Plot 2, and PDP3: Pendang Plot 3.

TF is known as an indicator of heavy metal accumulation in plants or mobility of heavy metals in the soil and also quantifies the differences in the bioavailability of metal to plant. The TF value from soil to root (TF_Soil_), root to stem (TF_Root_), and stem to grain (TF_Stem_) are presented in Figure 3, Figure 4 and Figure 5, respectively. In Figure 3, the TF_Soil_ values for all studied metals and areas were less than 1 except for Cu in the YP1 area with a TF value of 2.25. Figure 4 shows the TF_Root_ values from root to stem in all studied areas. The translocation values from stem to grain (TF_Stem_) are presented in Figure 5; the highest TF_Stem_ was found in KPP1 for Cr (13.61), while the lowest was in PDP2 for Cd (0.25). The translocation factor in paddy plants was determined as an indicator of metal translocation from soil to plant. TF demonstrates the capacity of plant to store heavy metals in its upper part [47]. The higher the TF values, the more mobile or available the metals are in plants [48,49]. The highest TF_Soil_ value was Cu in YP3 (1.26), whereas the lowest was Cr in YP2 (0.02), which stipulates that Cu was more mobile from soil to the root of paddy plants. Plants have the ability of tolerance to high concentrations of heavy metals through their mechanism by restricting transport from root to leaf, accumulation in trichomes, exudates that can complex the heavy metals, the type of link between the element and cell wall component, production of intracellular compounds with chelating properties, and active pumping to the vacuoles. The highest Cu value in YP3 may have been caused by the contaminated soil from water irrigation. Moreover, the high concentration of Cu in roots of YP3 may influence the value of TF_Soil_. The TF_Soil_ for Cd calculated in the present study does not agree with a previous study by Rahimi et al. [47] and Singh et al. [36] where Cd was in maximal by TF_Soil_ >1, Cd occurs in nature with Zn, and Cd(II) is held weakly by the soil compared with other harmful cations [50]. The accumulation of high quantities of Cd^2+^ in rice roots shows that Cd^2+^ is more bioavailable to plants than other heavy metals, resulting in a higher biological absorption coefficient [51]. Therefore, Cd is poorly bioavailable from soil to root as a result of a TF_Soil_ value of less than 1. The TF_Root_ for Cu in KPP3, YP2, and YP3 was greater than 1 with values of 1.82, 1.25, and 1.40, respectively.

### 3.5. Health Risk Assessment (HRA) of Paddy Grain Ingestion

**Figure 6:**
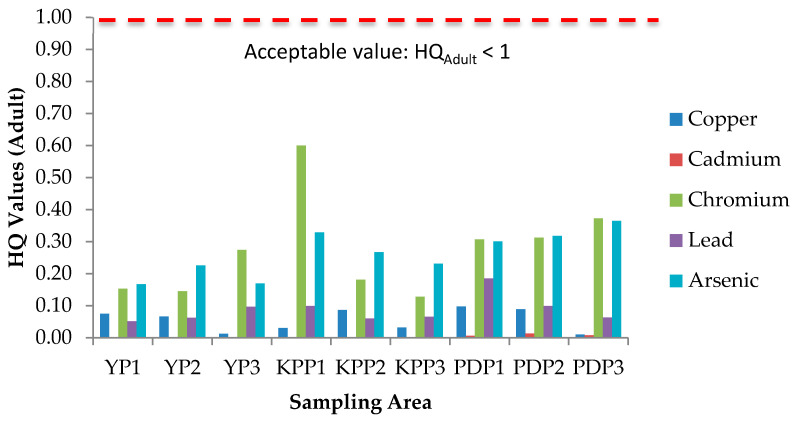
Hazard Quotient (HQ) values for adults in three plots of Yan, Kubang Pasu, and Pendang. YP1: Yan Plot 1, YP2: Yan Plot 2, YP3: Yan Plot 3, KPP1: Kubang Pasu Plot 1, KPP2: Kubang Pasu Plot 2, KPP3: Kubang Pasu Plot 3, PDP1: Pendang Plot 1, PDP2: Pendang Plot 2, and PDP3: Pendang Plot 3.

**Figure 7:**
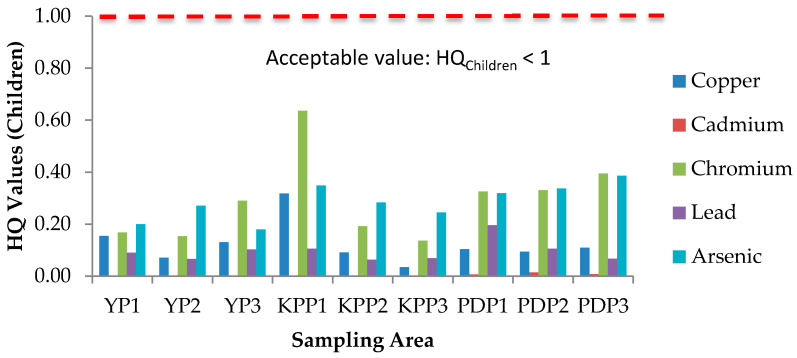
Hazard Quotient (HQ) values for children in three plots of Yan, Kubang Pasu, and Pendang. YP1: Yan Plot 1, YP2: Yan Plot 2, YP3: Yan Plot 3, KPP1: Kubang Pasu Plot 1, KPP2: Kubang Pasu Plot 2, KPP3: Kubang Pasu Plot 3, PDP1: Pendang Plot 1, PDP2: Pendang Plot 2, and PDP3: Pendang Plot 3.

**Figure 8:**
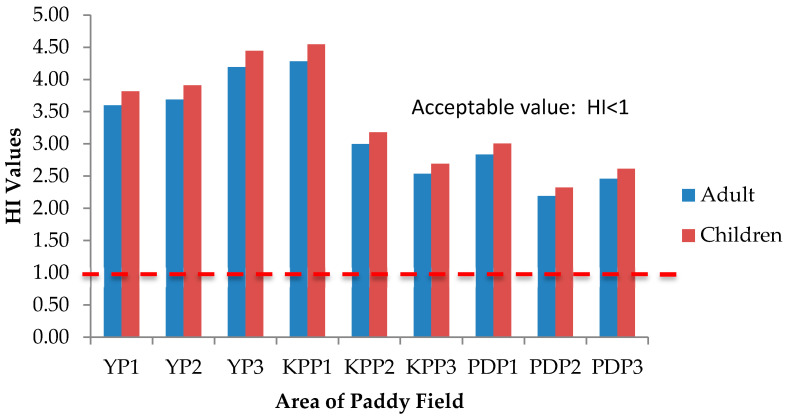
Hazard Index (HI) values of adults and children for three plots of Yan, Kubang Pasu, and Pendang. YP1: Yan Plot 1, YP2: Yan Plot 2, YP3: Yan Plot 3, KPP1: Kubang Pasu Plot 1, KPP2: Kubang Pasu Plot 2, KPP3: Kubang Pasu Plot 3, PDP1: Pendang Plot 1, PDP2: Pendang Plot 2, and PDP3: Pendang Plot 3.

**Figure 9:**
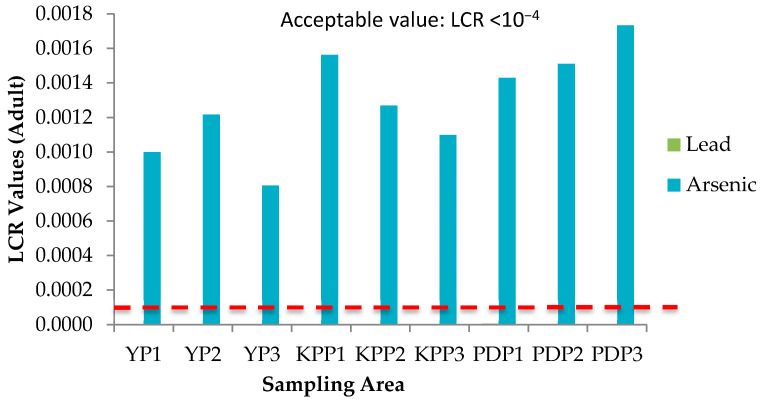
Lifetime cancer risk (LCR) values of adults and children for three plots of Yan, Kubang Pasu, and Pendang. YP1: Yan Plot 1, YP2: Yan Plot 2, YP3: Yan Plot 3, KPP1: Kubang Pasu Plot 1, KPP2: Kubang Pasu Plot 2, KPP3: Kubang Pasu Plot 3, PDP1: Pendang Plot 1, PDP2: Pendang Plot 2, and PDP3: Pendang Plot 3.

**Figure 10:**
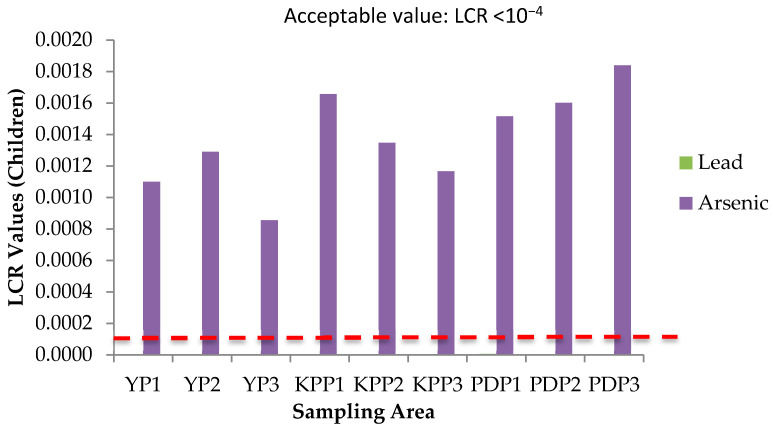
Lifetime cancer risk (LCR) values of adults and children for three plots of Yan, Kubang Pasu, and Pendang. YP1: Yan Plot 1, YP2: Yan Plot 2, YP3: Yan Plot 3, KPP1: Kubang Pasu Plot 1, KPP2: Kubang Pasu Plot 2, KPP3: Kubang Pasu Plot 3, PDP1: Pendang Plot 1, PDP2: Pendang Plot 2, and PDP3: Pendang Plot 3.

**Figure 11:**
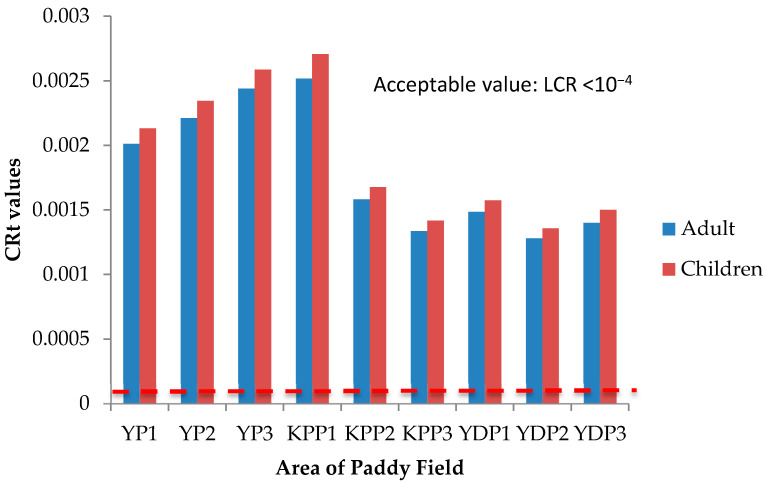
Total cancer risk (CRt) values of adults and children for three plots of Yan, Kubang Pasu, and Pendang. YP1: Yan Plot 1, YP2: Yan Plot 2, YP3: Yan Plot 3, KPP1: Kubang Pasu Plot 1, KPP2: Kubang Pasu Plot 2, KPP3: Kubang Pasu Plot 3, PDP1: Pendang Plot 1, PDP2: Pendang Plot 2, and PDP3: Pendang Plot 3.−

Health risks escalate following simultaneous exposure to heavy metals and other trace elements more than exposure to individual elements [51]. The International Agency for Research on Cancer (IARC) has classified arsenic (As) and lead (Pb) as both carcinogenic and non-carcinogenic elements, whereas cadmium (Cd), chromium (Cr), cobalt (Co), copper (Cu), iron (Fe), aluminum (Al), and zinc (Zn) as non-carcinogenic elements. The health risk assessment of each contaminant is normally based on the estimation of the risk level and is classified as carcinogenic or non-carcinogenic health hazards. To estimate the heavy metal contamination and potential carcinogenic and non-cancer health risk caused via ingestion of heavy metals in rice, Hazard Quotients (HQ), Hazard Index (HI), and Lifetime Cancer Risk (LCR) were used. The studied group in this study was adults and children. There is no potential of non-carcinogenic health risk since the HQ values for single heavy metal exposure for adults and children were less than 1, respectively (Figure 6 and Figure 7); however, there is a potential non-carcinogenic health risk for the combined heavy metal exposure for adults and children (Figure 8). The results are in line with the previous studies [52]. Meanwhile, for carcinogenic risk assessment, the acceptable range values of Lifetime Cancer Risk (LCR) recommended by USEPA [53] are in the range of 1 × 10^−6^ to 1 × 10^−4^, which means that a one to one hundred in a million chance of additional human cancer over a 70 year lifetime is considered as an acceptable or inconsequential risk. Figure 9 and Figure 10 demonstrate that the LCR values for Pb in all studied areas are within the acceptable value (LCR < 10^−4^), while As exceeds the acceptable value (LCR > 10^−4^) in accordance with the LCR values reported by Praveena and Omar [54]. Therefore, control measures should be put in place in order to reduce LCR values of carcinogenic metals by providing a safe source of rice to consumers. The CRt values (Figure 11) for all studied areas were higher than 10^−4^ indicating a high potential carcinogenic risk for both adults and children from rice consumption. Similar conclusions were reached in previous studies where the combination of heavy metals in rice was believed to pose a potential carcinogenic risk, approximately 82% of which was attributed to Cd [55]. Elevated Cd levels were found in soils and grain as a result of long-term mining activities in the Hunan province of China [45]. In this study, the LCR values for As and Cr contributed extensively to the high CRt value from which it may be inferred that the cancer risk may largely be attributed to these elements compared with Cd.

## 4. Conclusions

Heavy metal contamination in the environment is of increasing concern worldwide. An understanding of the sources of heavy metal contamination in agricultural soils and rice is indispensable in order to improve food safety by minimizing the possibility of food insecurity as well as reducing human health problems. In the current study, the concentration of heavy metals, namely As, Cd, Pb, Cu, and Cr, were determined in paddy soils and plants from the Yan, Kubang Pasu, and Pendang areas of Malaysia. Heavy metals accumulated more in roots than any other parts of the paddy. All heavy metals in paddy soils and rice grain were present at concentrations below the maximum permissible limit recommended by the Chinese Environmental Quality Standard for Soils, grade II (GB15618-2018), European standards agriculture soils, Malaysian Food Regulation 1985, and CODEX standard. However, some of the toxic metals studied, such as As and Cr, accumulated in rice grain, being potentially hazardous to human health. The concentration of individual heavy metals in paddy plants and soil determined in this study provide baseline data, and such studies should be extended to cover water quality and air quality monitoring. Moreover, assessment of human exposure to heavy metals through in vitro studies will clarify the total burden of environmental contamination.

## Figures and Tables

**Table 1:** Concentration of heavy metals in paddy soils from the Kedah area.

Area of Paddy Field	Concentration of Heavy Metals (µg/kg)				
	Arsenic (As)	Cadmium (Cd)	Lead (Pb)	Copper (Cu)	Chromium (Cr)
YP1	442.24 ± 53.18 ^a^	13.85 ± 1.87 ^a^	858.66 ± 29.39 ^b^	413.38 ± 2.10 ^b^	1508.10 ± 10.10 ^b^
YP2	497.99 ± 33.51 ^a^	12.74 ± 0.45 ^a^	869.99 ± 17.23 ^b^	489.84 ± 9.99 ^a^	1702.70 ± 30.40 ^a^
YP3	412.56 ± 7.02 ^a^	13.78 ± 1.28 ^a^	964.59 ± 23.07 ^a^	380.22 ± 8.60 ^c^	1083.50 ± 17.90 ^c^
KPP1	600.78 ± 11.61 ^a^	152.03 ± 1.50 ^a^	1107.60 ± 10.40 ^a^	345.06 ± 25.20 ^a^	1084.30 ± 6.60 ^b^
KPP2	618.23 ± 16.55 ^a^	119.86 ± 0.75 ^b^	857.30 ± 17.80 ^b^	364.53 ± 19.98 ^a^	1197.10 ± 27.30 ^a^
KPP3	468.28 ± 8.91 ^b^	80.27 ± 1.11 ^c^	744.90 ± 23.90 ^c^	283.24 ± 15.82 ^b^	983.60 ± 10.10 ^c^
PDP1	611.40 ± 9.72 ^a^	24.26 ± 0.77 ^a^	1063.90 ± 29.10 ^b^	385.21 ± 14.97 ^b^	1071.20 ± 26.50 ^a^
PDP2	455.77 ± 13.58 ^c^	17.03 ± 1.27 ^b^	1157.30 ± 14.60 ^a^	528.89 ± 11.78 ^a^	1100.10 ± 13.30 ^a^
PDP3	547.34 ± 13.47 ^b^	9.87 ± 0.77 ^c^	824.90 ± 26.50 ^c^	356.81 ± 4.85 ^b^	781.20 ± 7.80 ^b^
GB15618-2018 ^A^	25,000	300	300,000	-	300,000
EU Standards ^B^	-	3000	300,000	140,000	-

Note: Results are presented as Mean ± SD for concentration of heavy metals in paddy soils from the Kedah area. ^a–c^ Values in columns with different superscripts between plots of each area refer to statistical significance at *p* < 0.05; ^A^ maximum allowable concentration of heavy metals in soil, recommended by the Chinese Environmental Quality Standard for Soils, grade II (GB15618-2018); ^B^ European standards agriculture soils. YP1: Yan Plot 1, YP2: Yan Plot 2, YP3: Yan Plot 3, KPP1: Kubang Pasu Plot 1, KPP2: Kubang Pasu Plot 2, KPP3: Kubang Pasu Plot 3, PDP1: Pendang Plot 1, PDP2: Pendang Plot 2, and PDP3: Pendang Plot 3.

**Table 2:** Concentration of heavy metals in paddy plant parts from the Yan area.

Paddy Plant Parts	Plot	Concentration of Heavy Metals (µg/kg)				
		Arsenic (As)	Cadmium (Cd)	Lead (Pb)	Copper (Cu)	Chromium (Cr)
Root	YP1	323.60 ± 38.60 ^a^	2.40 ± 0.30 ^a^	226.90 ± 16.90 ^a^	996.60 ± 307.40 ^a^	145.80 ± 28.20 ^a^
	YP2	195.30 ± 13.70 ^b^	1.80 ± 0.10 ^b^	134.20 ± 8.00 ^b^	55.00 ± 2.30 ^b^	120.10 ± 12.30 ^a^
	YP3	98.60 ± 7.40 ^c^	2.00 ± 0.20 ^ab^	192.90 ± 17.40 ^a^	48.70 ± 4.30 ^b^	148.70 ± 12.10 ^a^
Stem	YP1	79.80 ± 6.60 ^a^	2.70 ± 0.20 ^a^	39.10± 11.00 ^a^	100.10 ± 4.70 ^a^	42.70 ± 0.90 ^a^
	YP2	75.70 ± 0.90 ^a^	1.00 ± 0.02 ^b^	14.70 ± 1.90 ^b^	68.90 ± 3.00 ^b^	35.10 ± 1.70 ^b^
	YP3	50.20 ± 1.40 ^b^	0.60 ± 0.10 ^c^	19.90 ± 4.50 ^b^	68.30± 17.70 ^b^	36.40 ± 1.30 ^b^
Leaf	YP1	30.40 ± 5.70 ^a^	1.60 ± 0.20 ^a^	48.8 ± 7.50 ^a^	181.30 ± 20.50 ^a^	14.50 ± 2.10 ^a^
	YP2	39.30 ± 2.70 ^a^	0.50 ± 0.10 ^b^	22.80 ± 1.70 ^b^	94.10 ± 7.00 ^b^	9.40 ± 1.70 ^b^
	YP3	6.90 ± 0.40 ^b^	0.20 ± 0.02 ^c^	16.40 ± 0.20 ^b^	107.70 ± 8.00 ^b^	18.40 ± 1.70 ^a^
Grain	YP1	80.10 ± 1.10 ^a^	0.20 ± 0.20 ^a^	23.50 ± 1.90 ^a^	69.40 ± 7.90 ^b^	45.40 ± 4.30 ^b^
	YP2	63.10 ± 1.70 ^a^	1.00 ± 0.10 ^a^	30.50 ± 2.60 ^a^	89.40 ± 6.00 ^b^	67.40 ± 8.60 ^b^
	YP3	53.10 ± 2.50 ^b^	0.80 ± 0.10 ^a^	36.40 ± 4.90 ^a^	128.00 ± 2.90 ^a^	85.70 ± 10.90 ^a^

Note: Results are presented as Mean ± SD for concentration of heavy metals in paddy plant parts from the Yan area. ^a–c^ Values in columns with different superscripts between plots of each area refer to statistical significance at *p* < 0.05; ^A^ maximum allowable concentration of heavy metals in rice grain permitted by Malaysian Food Regulation 1985; ^B^ maximum allowable concentration of heavy metals in rice grain permitted by CODEX standard. YP1: Yan Plot 1, YP2: Yan Plot 2, YP3: Yan Plot 3.

**Table 3:** Concentration of heavy metals in paddy plant parts from the Kubang Pasu area.

Paddy Plant Parts	Plot	Concentration of Heavy Metals (µg/kg)				
		Arsenic (As)	Cadmium (Cd)	Lead (Pb)	Copper (Cu)	Chromium (Cr)
Root	KPP1	140.30 ± 3.00 ^c^	3.00 ± 0.20 ^a^	297.00 ± 3.60 ^a^	71.50 ± 5.80 ^ab^	76.30 ± 8.30 ^a^
	KPP2	261.70 ± 8.30 ^a^	1.30 ± 0.70 ^b^	92.90 ± 5.50 ^c^	59.40 ± 4.60 ^b^	23.60 ± 1.10 ^b^
	KPP3	202.30 ± 17.20 ^b^	2.40 ± 0.10 ^a^	270.00 ± 10.50 ^b^	77.10 ± 6.80 ^a^	74.30 ± 5.70 ^a^
Stem	KPP1	16.60 ± 2.40 ^a^	0.20 ± 0.03 ^a^	5.70 ± 1.30 ^b^	44.60 ± 8.20 ^b^	9.00 ± 1.50 ^b^
	KPP2	16.00 ± 2.30 ^a^	0.20 ± 0.02 ^b^	3.60 ± 0.90 ^b^	38.10 ± 8.50 ^b^	8.70 ± 1.20 ^b^
	KPP3	17.50 ± 0.70 ^a^	1.80 ± 0.10 ^b^	11.50 ± 2.00 ^a^	140.30 ± 18.00 ^a^	15.70 ± 2.00 ^b^
Leaf	KPP1	25.80 ± 2.30 ^b^	1.00 ± 0.10 ^a^	10.60 ± 0.90 ^a^	56.20 ± 5.80 ^a^	8.20 ± 1.40 ^a^
	KPP2	32.40 ± 2.10 ^a^	0.10 ± 0.02 ^b^	9.00 ± 6.80 ^a^	61.80 ± 9.40 ^a^	5.30 ± 0.90 ^b^
	KPP3	29.80 ± 0.70 ^ab^	0.20 ± 0.10 ^b^	13.50 ± 6.90 ^a^	54.60 ± 3.30 ^a^	4.20 ± 0.50 ^b^
Grain	KPP1	94.10 ± 6.60 ^b^	0.60 ± 0.10 ^a^	69.50 ± 21.40 ^a^	175.98 ± 51.50 ^a^	96.20 ± 7.10 ^b^
	KPP2	99.50 ± 5.90 ^ab^	1.30 ± 1.90 ^a^	37.30 ± 47.60 ^a^	92.30 ± 40.90 ^a^	97.70 ± 2.20 ^b^
	KPP3	114.20 ± 9.40 ^a^	0.80 ± 0.20 ^a^	23.60 ± 6.10 ^a^	107.10 ± 63.80 ^a^	116.60 ± 3.70 ^a^
Food Regulation 1985 ^A^		1000	400	2000	-	-
CODEX standard ^B^		200	400	200	20,000	-

Note: Results are presented as Mean ± SD for concentration of heavy metals in paddy plant parts from the Kubang Pasu area. ^a–c^ Values in columns with different superscripts between plots of each area refer to statistical significance at *p* < 0.05; ^A^ maximum allowable concentration of heavy metals in rice grain permitted by Malaysian Food Regulation 1985; ^B^ maximum allowable concentration of heavy metals in rice grain permitted by CODEX standard. KPP1: Kubang Pasu Plot 1, KPP2: Kubang Pasu Plot 2, KPP3: Kubang Pasu Plot 3.

**Table 4:** Concentration of heavy metals in paddy plant parts from the Pendang area.

Paddy Plant Parts	Plot	Concentration of Heavy Metals (µg/kg)				
		Arsenic (As)	Cadmium (Cd)	Lead (Pb)	Copper (Cu)	Chromium (Cr)
Root	PDP1	100.20 ± 5.40 ^b^	1.50 ± 0.20 ^b^	143.80 ± 10.9 ^b^	116.50 ± 9.20 ^a^	91.3 ± 7.80 ^a^
	PDP2	273.50 ± 18.60 ^a^	0.90 ± 0.70 ^c^	103.60 ± 7.50 ^c^	52.00 ± 5.80 ^c^	30.70 ± 2.40 ^b^
	PDP3	250.50 ± 7.80 ^a^	3.40 ± 0.01 ^a^	178.70 ± 5.70 ^a^	89.60 ± 4.90 ^b^	88.30 ± 0.80 ^a^
Stem	PDP1	56.10 ± 6.00 ^a^	0.30 ± 0.10 ^b^	5.20 ± 0.80 ^a^	54.90 ± 3.40 ^ab^	13.80 ± 0.70 ^b^
	PDP2	16.50 ± 3.00 ^b^	0.20 ± 0.10 ^b^	5.10 ± 1.20 ^a^	43.40 ± 0.40 ^b^	6.70 ± 0.60 ^c^
	PDP3	13.60 ± 1.70 ^b^	0.50 ± 0.04 ^a^	3.30 ± 0.04 ^a^	67.80 ± 11.60 ^a^	27.10 ± 4.50 ^a^
Leaf	PDP1	66.60 ± 2.90 ^a^	4.00 ± 0.10 ^a^	14.60 ± 1.00 ^a^	5061.00 ± 732.30 ^a^	138.00 ± 18.30 ^a^
	PDP2	43.70 ± 3.10 ^b^	5.00 ± 0.10 ^a^	14.00 ± 0.90 ^a^	440.30 ± 25.60 ^b^	18.60 ± 1.00 ^b^
	PDP3	70.00 ± 4.10 ^a^	4.00 ± 0.02 ^a^	6.80 ± 1.40 ^b^	765.30 ± 199.40 ^b^	24.50 ± 2.60 ^b^
Grain	PDP1	102.90 ± 3.00 ^a^	0.20 ± 0.10 ^a^	037.20 ± 4.70 ^a^	43.70 ± 3.70 ^a^	95.90 ± 6.60 ^a^
	PDP2	83.60 ± 9.90 ^ab^	0.20 ± 0.00 ^a^	22.40 ± 7.40 ^a^	89.90 ± 19.50 ^b^	56.60 ± 12.30 ^b^
	PDP3	72.40 ± 9.30 ^b^	0.20 ± 0.10 ^a^	24.05 ± 6.30 ^a^	33.20 ± 6.00 ^b^	40.10 ± 9.20 ^b^
Food Regulation 1985 ^A^		1000	400	2000	-	-
CODEX standard ^B^		200	400	200	20,000	-

Note: Results are presented a Mean ± SD for concentration of heavy metals in paddy plant parts from the Pendang area. ^a–c^ Values in columns with different superscripts between plots of each area refer to statistical significance at *p* < 0.05; ^A^ maximum allowable concentration of heavy metals in rice grain permitted by Malaysian Food Regulation 1985; ^B^ maximum allowable concentration of heavy metals in rice grain permitted by CODEX standard. PDP1: Pendang Plot 1, PDP2: Pendang Plot 2, and PDP3: Pendang Plot 3.
